# Exploring the human brain: spatial transcriptomics challenges and approaches in post-mortem analysis

**DOI:** 10.1093/brain/awaf452

**Published:** 2025-12-05

**Authors:** Sean Chang, Christelle El Haj, Jan Mulder, Lipin Loo, Asheeta A Prasad

**Affiliations:** Discipline of Neuroscience, School of Medical Sciences, Faculty of Medicine and Health, University of Sydney, Camperdown, NSW 2050, Australia; Charles Perkins Center, University of Sydney, NSW 2006, Australia; Discipline of Neuroscience, School of Medical Sciences, Faculty of Medicine and Health, University of Sydney, Camperdown, NSW 2050, Australia; Department of Neuroscience, Karolinska Institute, 171 77 Stockholm, Sweden; Charles Perkins Center, University of Sydney, NSW 2006, Australia; School of Life and Environmental Sciences, University of Sydney, NSW 2006, Australia; Discipline of Neuroscience, School of Medical Sciences, Faculty of Medicine and Health, University of Sydney, Camperdown, NSW 2050, Australia; Charles Perkins Center, University of Sydney, NSW 2006, Australia

**Keywords:** human brain, spatial transcriptomics, post-mortem analysis

## Abstract

Over the past century, studying the human brain has been one of the most complex and enduring biological challenges. Initial approaches, ranging from gross neural anatomy to cellular subtype organization, have significantly advanced our understanding of the intricate structure of the human brain. Recent innovations in spatial transcriptomic technologies offer high-resolution insights into mRNA expression at single-cell or even subcellular resolution. Developing a greater understanding of the spatial expression of genes in specific cell types in the human brain can provide additional insights into their functions and underlying mechanisms that influence neurological disease states. Although these tools have been highly successful in rodent and non-human primate brains, analysis of the human brain has several specific challenges. In this review, we initially provide a comparison of spatial transcriptomic tools, followed by a summary of studies using these tools in human brains, and finally, we discuss the associated challenges and opportunities. The guidelines should enable researchers to address the challenges of using new spatial transcriptomic technologies to analyse complex organs, such as the human brain.

## Teaser

The molecular architecture of the brain underpins its organisation, function, and vulnerability to disease. New spatial transcriptomic tools allow visualisation and analysis of more than 100 genes at once. Chang *et al.* review the challenges and opportunities associated with applying these tools to post-mortem human brain tissue.

## Introduction

The neuroarchitecture of the human brain fundamentally underpins complex human functions. This highly heterogeneous organ consists of diverse anatomical regions, each composed of various cell types of variable population sizes. Such diversity is facilitated by variations in gene expression within different cell types. Our early understanding of neural organization stems from classical histological techniques, such as Golgi staining, which enabled insight into the microstructure of the human brain.^1,2^ The recognition of regional specializations within the brain following Brodmann’s work enhanced our understanding of brain structure and functioning.^3^ Spatial analysis studies targeting protein detection have been fundamental and have increased our knowledge of human brain organization and pathological characterization.

**Figure 1:**
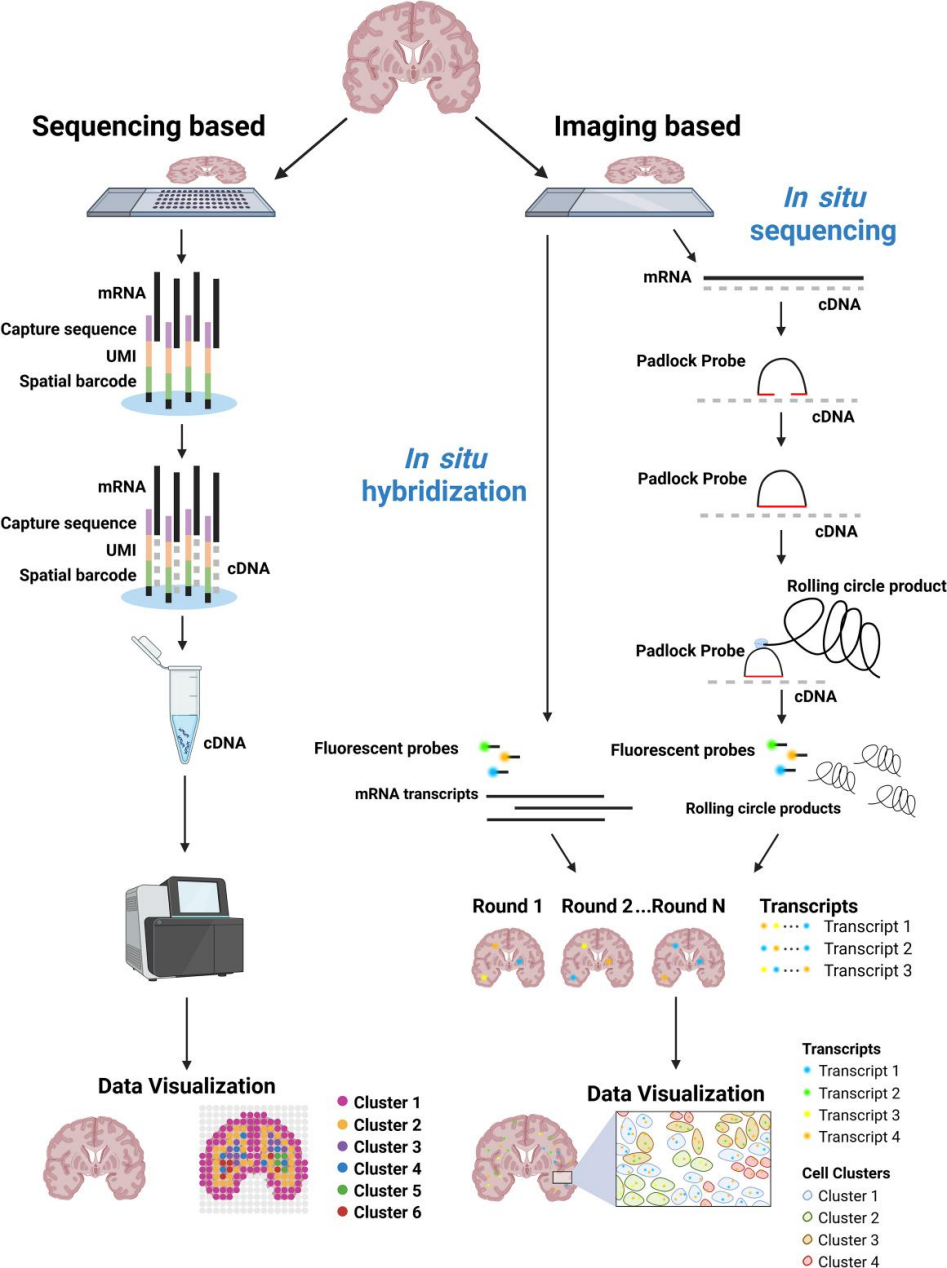
**Schematic of spatial transcriptomic types.** Sequencing-based ST relies on specialized slides that have an array of ‘spots’ and contain oligonucleotides designed to capture mRNA transcripts. After permeabilization of the tissue, mRNA transcripts are captured by oligonucleotides on the specialized slide. These are then reverse transcribed, along with a UMI and ‘spatial barcode’, to generate a cDNA ‘library’ containing all the captured transcripts and their associated spatial information. These then undergo next-generation sequencing to uncover the identity and spatial origin of the mRNA transcripts present. *In situ* hybridization ST relies on fluorescent probes added to the tissue of interest. Sequential rounds of fluorescent probe hybridization then occur, with probes hybridizing to target transcripts. This fluorescent signal is then read out to identify the mRNA transcripts present in the tissue. *In situ* sequencing ST first creates cDNA copies of the mRNA transcripts within a tissue. Padlock probes are added and hybridize to cDNA. Probe ligation occurs, creating a round template from which RCA can occur. RCA occurs, producing ‘rolling circle products’ that contain many copies of the padlock probe. Sequential rounds of fluorescent probe hybridization occur, with probes hybridizing to rolling circle products. This fluorescent signal is then read out to identify the mRNA transcripts present in the tissue. (Note that variations on these main methods exist). FF = fresh frozen; FFPE = formalin fixed paraffin embedded; RCA = rolling circle amplification; ST = spatial transcriptomics; UMI = unique molecular identifier. Created in BioRender. Prasad, A. (2025) https://BioRender.com/5eol2o5.

The advent of RNA sequencing technologies has increased the analysis of multiple gene expressions exponentially. Bulk RNA sequencing enabled a first look into the complete transcriptome of human tissues, allowing for changes in gene expression to be measured, including abnormal alterations in disease states.^4^ Further technological development led to increased resolution at the single-cell level, known as scRNA-seq, allowing researchers to identify the molecular signatures of cell types within tissues more precisely. Although this approach has led to many discoveries, the harsh dissociation required to generate a single-cell suspension can result in the biased selection of more robust cells, such as oligodendrocytes and neurons, over glial cells. Many researchers use single-nucleus RNA sequencing (snRNA-seq) instead to circumvent this issue, but this approach does not profile cytoplasmic mRNA. More recently, spatial transcriptomics (ST) has emerged as the new frontier in human neuroscience.^5-7^ With high-resolution capabilities of spatial profiling of the transcriptome, it enables an unprecedented level of insight into the organization and microcellular environment of diverse cell types in the brain without the need for single-cell/nucleus dissociation. We will initially provide an overview of the vast variety of ST platforms, including imaging- and sequencing-based ST (Fig. 1), before discussing the challenges associated with post-mortem brain profiling.

## Sequencing-based spatial transcriptomics

Sequencing-based ST relies on capturing mRNA transcripts from biological tissues while preserving information on spatial location. These captured mRNA transcripts are used to generate a cDNA library, which undergoes next-generation sequencing to reveal the spatial expression of genes in the analysed tissue. The capture of mRNA transcripts is commonly achieved using specialized slides, upon which the biological tissue of interest is placed. These slides contain many spots in an array, with each of these spots containing oligonucleotides designed to capture mRNA. These oligonucleotides have three main component sequences: one to identify the region (known as the ‘spatial barcode’); one that identifies the molecule (known as a unique molecular identifier, or UMI, used for quantifying unique transcripts); and one to capture the mRNA transcripts [for example, a poly(dT) sequence to capture transcripts by binding to their poly(A) tails]. Tissue permeabilization results in mRNA transcripts being released to be captured by oligonucleotides on the specialized slide. Reverse transcription then enables the captured mRNA to be converted into cDNA, along with the relevant spatial barcode and UMI sequence. These products undergo sequencing before bioinformatics processing to assign gene labels and locate the ‘spot’ on the slide from which it originated. Thus, unlike imaging-based ST methods, which have an upper limit on the number of transcripts that can be detected simultaneously, sequencing-based platforms provide less-biased whole-transcriptome coverage because all captured transcripts can, in principle, be sequenced.

This approach is adopted by most sequencing-based ST platforms, with some variations between platforms (see Table 1). A point of variation lies in the size and distance between spots on the slide, which results in differences in the spatial resolution obtainable by each platform. Other alternative transcript capture methods incorporate oligonucleotide probes in their design, which hybridize with target transcripts within the tissue before being captured on slides. This extra step can improve the sensitivity of the ST platform^17^ and/or make it compatible with formalin-fixed paraffin-embedded (FFPE) tissue, which typically has more fragmented transcripts than fresh frozen tissues, posing a challenge to mRNA capture in this tissue type. A caveat of this approach is that it is potentially more biased, because any transcripts that are not targeted by probes cannot be detected by this method, although given that, theoretically, the whole transcriptome is targeted by these probes, this might not pose an issue.

**Table 1:** Comparison between some common sequencing-based spatial transcriptomics platforms with examples of their use in human brains

ST platform	Tissues compatible	Resolution	Capture area	Examples in human brains	Transcripts detectable	Sensitivity	Additional notes
Visium (formerly ST)	FF and FFPE	55 μm spots, with 100 μm between spots	6.5 mm × 6.5 mm or 11 mm × 11 mm	Chen *et al*.,^8,9^ Zeng *et al*.,^10^ Simard *et al*.,^11^ Maynard *et al*.,^12^ Huuki-Myers *et al*.,^13^	Untargeted; whole transcriptome^a^	Lower than Visium HD	–
Visium HD	FF and FFPE	2 mm × 2 μm	6.5 mm × 6.5 mm	–	Untargeted; whole transcriptome^a^	Moderate to high	Bin size is higher at 8 mm × 8 μm. Greater sensitivity than Visium
STEREO-seq	FF (FFPE with STERO-seq OMNI)	0.2 μm spots, with 0.5 μm between spots	Many options, up to 13 mm × 13 cm	Li *et al*.,^14^ Gong *et al*.^15^	Untargeted; whole transcriptome	High	High resolution, largest capture area currently available
Slide-seq (Curio Seeker)	FF	10 µm	3 mm × 3 mm or 10 mm × 10 mm	Kamath *et al*.^16^	Untargeted; whole transcriptome	Low	–
Slide-seq V2	FF	10 µm	0.3 mm diameter	–	Untargeted; whole transcriptome	Moderate to high	Greater capture efficiency and sensitivity than slide-seq

FF = fresh frozen; FFPE = formalin fixed paraffin embedded; ST = spatial transcriptomics.

^a^Visium HD and Visium FFPE use targeted probes that cover the whole transcriptome; in a sense, it is ‘targeted’ but for the entire transcriptome.

Another concern is that the size of their capture areas limits many sequencing-based ST platforms that rely on specialized slides. This limits the maximum size of tissues that can be analysed. Some technology providers can produce customized larger capture areas at an additional cost. Despite this limitation, the less biased approach of sequencing-based ST enables profiling of the entire transcriptome, well suited for discovery projects, in contrast to methods based on gene panels.

## Imaging-based spatial transcriptomics

### *In situ* hybridization

Imaging-based ST platforms rely on the hybridization of fluorescent oligonucleotide probes to target mRNA transcripts, localizing specific transcripts to spatial locations within the tissue. The mRNA detection approach varies based on the platform. This includes using a specific individual or combination of fluorophores to identify unique transcripts.

The advantages of *in situ* hybridization (ISH)-based and other imaging-based platforms often include higher resolution and transcript sensitivity than sequencing-based ST. As such, these platforms are often suitable for more targeted, detailed validations of preliminary investigations following unbiased sequencing-based platforms. However, a key limitation of ISH-based platforms is their inability to profile the entire transcriptome. Furthermore, they suffer from imaging-based limitations, such as optical crowding, which might necessitate changes in the set of probes used for analysis.

A key consideration for ISH-based platforms is the number of transcripts detectable by the platform, referred to as the ‘plexity’. Recent advancements, such as those from Nanostring and 10× Genomics, have significantly improved plexity, allowing for the profiling of hundreds to thousands of transcripts. Although some platforms might still profile fewer transcripts, the selection of probes has expanded, reducing the risk of selective bias. By choosing platforms with larger probe sets, researchers can now detect a broader range of transcripts, enhancing the robustness of their analyses.

### *In situ* sequencing

This method involves generating cDNA copies of target mRNA transcripts *in situ*. These are then targeted with padlock probes, with sequences on their 3′ and 5′ ends that hybridize to cDNA. Upon successful hybridization, these ends ligate together, enabling the rolling circle amplification (RCA) of the padlock probes to occur. Fluorescent probes complementary to the RCA products enable these cDNA copies to be imaged and identified. An alternative to padlock probes is circularization of the cDNA for RCA. This method is untargeted, allowing whole-genome coverage for *in situ* sequencing (ISS) platforms using it.

## Challenges and solutions of spatial transcriptomic platforms

Some concerns with ST platforms include differences in sensitivity between platforms (see Table 2). This can pose a particular challenge in detecting genes of interest that are rare or have low expression and in comparing datasets generated from these different platforms. Integrating sc/snRNA-seq data with ST data is a possible solution, allowing for the imputation of cell types and gene expressions while preserving spatial information.

**Table 2:** Comparison between common imaging-based spatial transcriptomics platforms, with examples of their use in human brains

ST platform	Tissues compatible	Capture area	Transcripts detectable	Examples in human brains	Performance caveats
***In situ* hybridization (ISH)-based platforms**					
smFISH	FF	N/A	1–4	Maynard *et al*.^12^	Low multiplex capability
RNAscope	FF and FFPE	20 mm × 20 mm	1–4	Simard *et al*.,^11^ Huuki-Myers *et al*.,^13^ Chen *et al*.^9^	Sold as a reagent kit, with low multiplex capability
MERFISH	FF and FFPE	20 mm × 15 mm	∼10 000	Fang *et al*.,^18^ Kim *et al*.^19^	Requires specialized slides, optical crowding and long workflow
seqFISH	FF	N/A	∼250	–	Optical crowding and long workflow^20^
seqFISH+	FF	N/A	∼10 000	–	Higher multiplexing capability version of seqFISH, but with lower sensitivity
osmFISH	FF	N/A	33	–	Long imaging time, with potential RNA loss per cycle^21^
CosMx	FF and FFPE	15 mm × 20 mm	Whole transcriptome	Harwood *et al*.^22^	Offers the ability also to spatially profile ≤100 proteins
GeoMx	FF and FFPE	Dependent on user aims	Up to whole transcriptome	Harwood *et al*.,^22^ Goralski *et al*.,^23^ Kopić *et al*.^24^	User-defined ‘region of interest’ that will be targeted by GeoMx. Lower sensitivity in smaller regions of interest
***In situ* sequencing (ISS)-based platform**					
ISS	FF or FFPE	N/A	300	Chen *et al*.^9^	–
STARmap	FF	N/A	∼1020	–	Low detection efficiency
FISSEQ	FF or FFPE	N/A	Untargeted; whole transcriptome	–	Very low detection efficiency. Under-represents highly expressed genes^25^
ExSeq	FF or FFPE	N/A	Targeted: 42 Untargeted: 1000	–	Nanoscale resolution
Xenium	FFPE or FF	12 mm × 24 mm	5000	–	Require specialized slides
HybISS	FF or FFPE	N/A	∼120	Langseth *et al*.^26^	–

Transcripts detectable refers to the maximum number of transcripts detectable. FF = fresh frozen; FFPE = formalin fixed paraffin embedded; N/A = not assessed; ST = spatial transcriptomics.

Although sequencing-based ST platforms offer whole-transcriptome coverage (see ‘*Sequencing-based spatial transcriptomics*’), they typically have low sensitivity, especially in comparison to most imaging-based ST platforms. However, variations in the sensitivity of sequencing-based ST do exist.^17^ Another important factor to consider is that ST platforms more broadly have been shown to have different sensitivities depending on the tissue being analysed (e.g. brain versus eye tissue), such that even in the same tissue, the measured transcript abundance and location can vary depending on the ST platform used.^17,27,28^ On a related note, ST platforms have also shown different sensitivities for different transcripts.^17,27,29^ These factors might be important when designing experiments using ST platforms or comparing datasets. Additionally, not all ST methods are compatible with FFPE tissue, a common format used to preserve post-mortem human brain tissues (Table 2). This can limit the ST platforms or tissues used for investigations.

Despite these challenges, ST platforms have shown great promise in brain profiling, offering valuable insights into the spatial organization and function of brain tissues. Next, we will summarize the key ST brain studies conducted to date, highlighting their contributions to our understanding of brain biology.

## Application of spatial transcriptomics in post-mortem human brains

This ability of ST has been leveraged increasingly over recent years as the technology continues to mature and develop, growing in accessibility and capability. This has led to remarkable studies pushing forwards the envelope of current understanding in neuroscience in areas such as human neurodevelopment, Alzheimer’s disease (AD) and Lewy-body-related pathologies.

### Human neurodevelopment

The genesis of the human brain is a highly complex orchestration involving a massive proliferation of cells and a tremendous expansion of brain volume. Crucially, this developmental phase is driven by high levels of differentiation as pluripotent stem cells, such as oligodendrocyte progenitor cells, astrocyte progenitor cells and radial glial (RG) cells, begin to specialize into the various neuronal cell types that form the adult human CNS. The differentiation and cell proliferation are highly regionalized processes with specific spatial distribution and highly heterogeneous structure. Although traditional single-cell or bulk sequencing techniques can capture single-cell heterogeneity, the spatial context is lacking to identify the localization of these specific cell types. Furthermore, these methods are not well suited to isolate and capture molecular signatures of rare cell populations or states. As such, the ability of ST to spatially profile biological tissues becomes an essential feature to provide the necessary detail required for further insight into a highly complex and crucial developmental process.

A milestone study that used a combination of scStereo-seq, a sequencing-based ST platform, and scRNA-seq to construct a spatiotemporal ‘atlas’ of the developing human brain from 6 to 23 gestational weeks was conducted by Li *et al*.^14^ in 2023. This study revealed that the RG cells display regional heterogeneity and specific spatial distribution of subtypes. Such phenomena were additionally found to contribute to subsequent neuronal specification, specifically, two diencephalon-specific subtypes of RG cells were observed, with the population on the ventral portion developing mainly into GABAergic neurons and the population on the dorsal portion developing mainly into glutamatergic neurons. Additionally, RG subtypes in the ventral midbrain were associated with developing dopaminergic neurons. As such, through the use of ST, this study was able to probe deep into the early development of the human brain, revealing signalling interactions between cell types and the large degree of influence it has on their development and regionalization.

Neurogenesis in adult humans was initially reported in 1998^30^ and has remained a significant area of investigation since. Simard *et al*.^11^ investigated whether neurogenesis occurs in the adult human hippocampus using Visium, a sequencing-based ST platform, and validated the findings with RNAscope. Their results indicated that very little neurogenesis occurred in the adult hippocampus. They identified a potential local reserve of plasticity, in the form of a small population of immature granule neurons of predominantly GABAergic identity within the dentate gyrus of the hippocampus. This was speculated to provide a potential ‘buffer’ against age-related damage to the brain; however, the authors conceded that more work needed to be done to assess whether this is the case for humans.

### Alzheimer’s disease

Amyloid plaque pathology is a key hallmark of AD and is thought to be a potential cause of the disease.^31^ Chen *et al*.^9^ used RNAscope to analyse the human middle temporal gyrus, generating a spatial map of transcriptomic changes with increasing amyloid plaque pathology. Using the spatial information, the microenvironment around amyloid plaques could be identified and investigated, resulting in the discovery of 57 plaque-induced genes in the 100 μm area around the plaques, which the authors of the study proposed forms part of a coordinated cellular response to amyloid plaques in the brain. In another study, Gong *et al*.,^15^ using Stereo-seq in the human prefrontal cortex, identified significant disruptions to the laminar architecture and alterations of gene expression within the prefrontal cortex in late-stage AD.

Interestingly, the authors identified that a key gene, Death-associated protein kinase 1 (*DAPK1*), a regulator of neuronal apoptosis, was significantly enriched in cortical layers II and III in the moderate AD group compared with control and severe cases. Impaired signalling interactions were also noted, particularly in glutamate and neurexin (NRXN) signalling, which exhibited significant dysregulation in the moderate AD group compared with controls. This indicated compromised neural connectivity, potentially contributing to cognitive decline in AD. Furthermore, distinct stress response patterns were observed in neurons and their adjacent glial cells, with neuroprotective and amyloid-β clearance mechanisms found to be compromised in AD. These were considered key factors contributing to the degradation of neurons and deposition of amyloid-β plaques in AD.

These studies indicate the importance of studying cells within their environment and the potential to discover novel disease mechanisms that are difficult to dissect using bulk or single-cell approaches.

### Parkinson’s disease and Lewy-body dementia

In Parkinson’s disease (PD), Lewy body pathology is a diagnostic marker.^32^ However, a key question that remains to be answered is the effect of these Lewy bodies on the brain, in particular the surrounding microenvironment, and how this might impact nearby neurons and glial cells. Goralski *et al*.^23^ investigated cells and their environment in PD using a combination of a mouse model and post-mortem human brain tissue using the GeoMx Digital Spatial Profiler (DSP). Here, ST provided a unique advantage over sc/snRNA-seq. The latter techniques require that individual cells and nuclei be dissociated from tissue, hence features such as α-synuclein inclusions are prone to loss, whereas this limitation does not constrain ST. As such, this enabled the authors to probe for transcriptomic changes occurring around Lewy bodies. Staining was done for the pathology marker pS129 α-synuclein (pSyn) in post-mortem human brain tissue to identify neurons with α-synuclein pathology. ST analysis with GeoMx DSP revealed that neurons bearing α-synuclein inclusions demonstrated prominent changes in gene expression, indicating broad-ranging cellular dysfunction in neurons with inclusions. In total, 1422 genes were observed to be upregulated and 620 genes downregulated in these neurons. Of these, the top genes that were upregulated included those related to complement/cytokines, DNA/RNA integrity and ion channels. Top downregulated genes included those related to the synapse, metabolism, membrane signalling, endo-lysosome, ubiquitin–proteasome and the cytoskeleton.

Additionally, the authors identified neuronal populations in the cerebral cortex that were particularly at risk of or resistant to pathological changes. Specifically, using human samples, their results suggest that inhibitory neurons were less likely to contain α-synuclein inclusions, whereas excitatory neurons were more likely to possess these inclusions. Extending their investigation, they found that almost all pSyn inclusions occurred in excitatory glutamatergic neurons, whereas few were observed in inhibitory GABAergic neurons, using *SLC17A7* and *GAD1* markers, respectively.

### Amyotrophic lateral sclerosis

Although the specific vulnerability of motor neurons has been well established in amyotrophic lateral sclerosis, the application of ST has identified an interaction between brain connectivity and local vulnerability and a deeper understanding of previously known (*APOE*, *C9orf72*) genetic factors.^33-35^ Moreover, spatial analysis of amyotrophic lateral sclerosis brain tissue has led to the discovery of a new gene, *NOMO1*, expressed in affected motor neurons.^36^ Investigations into the common amyotrophic lateral sclerosis-linked genetic mutation in the *C9orf72* gene that severely impacts two key regions, the frontal cortex and cerebellum, have found dysfunction in a common cellular mechanism. The possibility to analyse changes in spatial enrichment of gene sets allowed the understanding of how a single genetic mutation can cause dysfunction in distinct brain regions.^34^

### Insight into other neurological disorders

ST analysis of temporal cortex samples from epileptic and non-epileptic subjects have revealed that specific changes in cell types occurred in distinct cortical layers. This finding has identified that epilepsy is not merely general neuron hyperexcitability, but rather a highly specific dysfunctional circuit.^37,38^ Other emerging studies in neuropsychiatric disorders have used ST technology successfully, including studies on post-traumatic stress disorder, major depressive disorder and schizophrenia.^39,40^ A landmark study recently integrated ST with genetic and epigenetic data from post-traumatic stress disorder post-mortem cases. Key findings aligned with previously linked genes associated with stress-related disorders, such as *FKBP5*, and also identified changes in glucocorticoid signalling, GABAergic transmission and neuroinflammation. Given that neuropsychiatric disorders manifest physical symptomology, this study represents a major breakthrough in understanding the change in neural responses in post-traumatic stress disorder.^39^

### Summary

**Figure 2:**
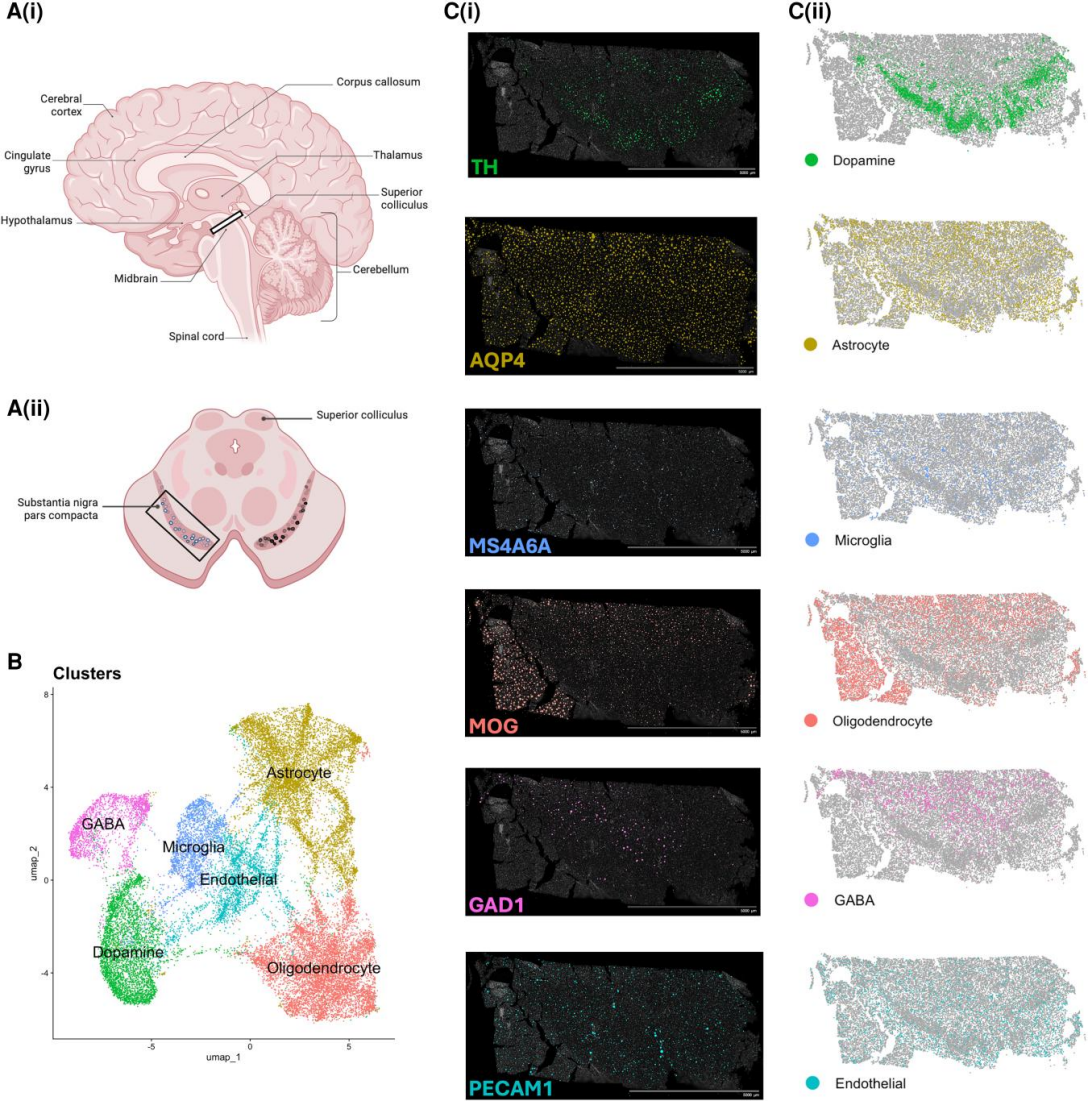
**Spatial transcriptomics on post-mortem human midbrain.** [**A**(**i**)] Sagittal view of the human brain highlighting the midbrain. Created in BioRender. Prasad, A. (2025) https://BioRender.com/5eol2o5. [**A**(**ii**)] Coronal view of the human midbrain outlining the substantia nigra pars compacta region selected for Xenium spatial transcriptomics profiling. (**B**) Different cell types within the substantia nigra were segregated into transcriptionally distinct clusters that were identified and visualized via principal component analysis and uniform manifold approximation and projection analysis in RStudio. [**C**(**i**)] Representative output of the Xenium spatial transcriptomics assay with cell-type-specific markers visualizing the spatial distribution of the cells using 10× Genomics’ Xenium Explorer 4 showing (from *top* to *bottom*): TH, tyrosine hydroxylase, a signature marker for dopamine neurons; *S100B*, a calcium-binding protein marker expressed primarily in astrocytes; *MS4A6A*, Membrane-spanning 4-Domains A6A, a specific microglia marker; *MOG*, myelin oligodendrocyte glycoprotein, an oligodendrocyte biomarker; *GAD1*, glutamate decarboxylase 1, a signature marker for GABAergic neurons; and *PECAM1*, platelet endothelial cell adhesion molecule-1, a biomarker for endothelial cells. [**C**(**ii**)] Visualization of clusters in the tissue sample shown in **C**(**i**) using the package RStudio Seurat. The cell clusters detected recreate similar expression patterns to their biomarkers in the software Xenium Explorer 4.

To advance understanding of human brain development and disease, ST technology reveals the transcriptional landscape of a tissue, uncovering cellular identities and diversity, clues to the microenvironments present and tissue heterogeneity, communicating complex organization and interactions. Data generated from the midbrain of a human post-mortem brain, from Dr Asheeta Prasad’s laboratory, are presented in Fig. 2. With the added spatial dimension provided by ST, such features can be investigated in much greater detail and precision than was previously possible. All in all, these studies showcase the tremendous potential of ST in human neuroscience. Although the number of studies currently might be fairly limited, the significant growth in uptake and the quality of the data gleaned from these studies highlight the disruptive potential of ST, pointing to a bright and exciting future for ST technologies in neuroscience.

## Challenges and limitations of spatial transcriptomics technologies in human brains

**Figure 3:**
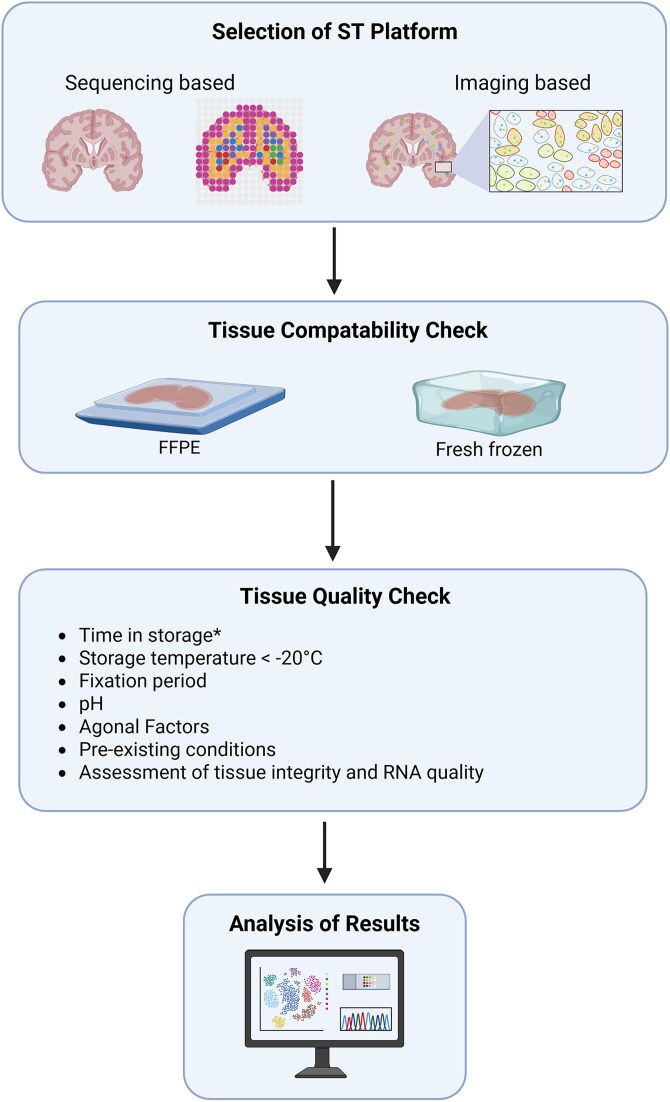
**Flowchart of considerations and pipeline for spatial transcriptomics (ST) experiments.** *Optimal time in storage is dependent on the temperature at which the tissue is stored, with lower temperatures allowing longer storage. See ‘FFPE samples’ or ‘Fresh frozen samples’ sections. Created in BioRender. Prasad, A. (2025) https://BioRender.com/5eol2o5.

Although ST is a powerful technique with enormous potential in human neuroscience, access to human brain tissues often poses the biggest challenge for ST studies on the human brain. Some of the associated challenges include limited availability of donors, pre-existing conditions and causes of death that cannot be controlled for in human post-mortem analysis. All these factors might potentially impact tissue integrity, and ST platforms rely on high RNA quality. Therefore, to apply the advancements of ST in neuroscience, it is important to understand the challenges associated explicitly with human post-mortem brains. Here, we provide a detailed look at some of the key considerations that should be taken into account when preparing to undertake a ST study using post-mortem human brain tissue (Fig. 3).

### Best practices for application of spatial transcriptomics in human brain studies

#### Consideration between fresh frozen and FFPE samples

For storage or brain banking of human brains, the post-mortem brain is typically divided into hemispheres. One hemisphere is then freshly isolated and frozen at −80°C, with tissues processed in this manner commonly referred to as fresh frozen tissues, and the other hemisphere is subjected to chemical fixation. Fixation using paraformaldehyde or formalin is the most common approach. The brain tissue is then embedded in paraffin and sectioned. The selection between fresh frozen and FFPE tissues presents unique strengths and weaknesses that must be evaluated for specific research questions, experimental design and the ST platform applied.

Although FFPE can preserve tissue integrity well, it may impact RNA integrity through RNA degradation^41-43^ and other chemical modifications^43,44^ as a result of the preservation process. The preservation method of paraffin embedding helps to preserve cytoarchitecture for the thin tissue sectioning process. This is extremely important for downstream cell segmentation and transcript localization. However, over-fixation or long-term storage in fixative before paraffin embedding reduces RNA yield. Owing to the larger size of human brains, fixation can be uneven in inner regions, leading to atrophy and reducing RNA transcript detection. On the other hand, fresh frozen (FF) tissue is fragile, potentially subject to freezing artefacts and sensitive to temperature. This limits the selection of ST platforms that can be used to assay the tissue and adversely affects the quality of the data collected from the available assays.

Alternatively, laboratories might opt to procure tissues with suitable collection criteria from brain banks. This includes specifications such as tissues that have been preserved for a shorter period, which are typically of higher quality. However, this can be difficult and impractical for many experimental designs.

##### Fresh frozen samples

If using frozen tissue, it is important to ensure that work is conducted swiftly and to maintain cool temperatures and avoid thawing where possible. Thawing of human cerebral tissue^45^ and various rodent tissues^46^ has been reported to cause severe RNA degradation, and deleterious effects on RNA^47,48^ and tissue^49^ quality have also been demonstrated in other areas and cell types after thawing. Fresh frozen tissue stored at −80°C has been found to retain high-quality RNA even up to 23 years in human brains.^50^ Other studies have also indicated that long-term storage has little to no effect on RNA quality in frozen human brains, recovering intact RNA after 19 years at −80°C.^51^ As such, time in storage is unlikely to be a significant factor for RNA quality if fresh frozen tissue is used, if it has been stored appropriately and not subjected to thawing events. Another key issue is the potential for tissue alterations attributable to freezing. However, if temperatures are cold enough (human liver tissue has previously been observed to have ice crystal formation after storage at −25°C for 7 years, but not for −80°C and below),^52^ this is unlikely to be affected by the duration in storage.

##### FFPE samples

For FFPE preserved tissue, where possible, tissues that have been in storage for shorter periods of time should be selected. RNA quality in FFPE tissue becomes progressively worse the longer the tissue is stored^53^ or fixed.^54^ Room temperature, where FFPE tissues are usually stored, leads to RNA degradation.^42,55-57^ Furthermore, long fixation times have been shown to affect transcript detectability adversely with ST in human brains^54^ and to reduce RNA^41^ and tissue quality. Autofluorescence was also found to be stronger with increased fixation time, increasing noise and making it more difficult to parse out true signals.^54^ Many methods have been developed to counter the effect of autofluorescence (see ‘Autofluorescence’ section). However, this can pose a challenge for large samples (e.g. whole brains), which require longer times in fixative in order for fixation to occur in the innermost regions. It has been reported previously that it takes between 20 and 46 days for a sufficient amount of fixative to diffuse into the innermost regions of the brain to cause fixation.^58^ Fixation for this long will be likely to result in over-fixation of outer layers, and additional degradation in innermost layers would be likely to occur owing to increased bacterial degradation and autolysis, caused by the delay in its fixation. If possible, tissues should be sectioned into sizes no larger than a standard tissue cassette for fixation, in order to avoid tissue or RNA degradation. Less than 5 mm is optimal, and the tissue should be fixed for at least 16–24 h. Additionally, it is recommended that FFPE tissues be kept below −20°C after fixation. However, it is important to note that the maximum age of FFPE tissue that can be used would depend largely on the ST platform, because the RNA quality tolerances for different platforms vary greatly.

#### Assessment of tissue integrity

Before conducting any assays, several forms of assessment should be done to assess the integrity and quality of the tissue samples being used. Generally, an initial step is haematoxylin and eosin staining to assess tissue integrity and the presence of artefacts and to confirm that the region of interest is contained within the sample. 4′,6-diamidino-2-phenylindole (DAPI) staining could also confirm good nuclei quality, because lower quality is associated with poorer results (10× recommendation sites for Xenium). Hence, standard staining processing can identify samples that are intact, with minimal damage.

#### Assessment of RNA quality

Determination of RNA quality is an important step that should be conducted before running any ST assays, because lower RNA quality will impact the quality and sensitivity of the data obtained from ST and reduce the chances of a successful assay. This includes issues with lower median transcripts per cell detected owing to greater RNA fragmentation in tissues with low RNA quality. Common approaches to assess RNA quality include the RNA integrity number (RIN) and percentage of RNA fragments generally >200 nucleotides. Yet, despite its widespread use, RIN is an unreliable measure of RNA quality^48^ and does not reflect expressional changes that can occur in tissue^59^ or mRNA integrity,^60^ because it is primarily a measurement of ribosomal RNA. Other methods, such as DV200,^61-63^ similar analyses using different fragment lengths as thresholds, such as DV100–300,^61^ or PCR-based methods^61,63^ have been shown to be better markers of RNA quality than RIN. Additionally, in the case of FFPE tissue, RIN values are typically low; however, they do not provide a good reflection of RNA quality.^63-65^ Owing to its ease and simplicity to conduct, combined with its good predictive power,^66^ we recommend using the DV200 assay to assess RNA quality in tissues prior to ST.

For the DV200 assay, a score of ≥30 is generally considered good.^65^ However, a good score will be dependent upon the ST platform being used, because they can have a higher or lower threshold than this. For instance, Visium FFPE is recommended to have a DV200 of ≥50%,^67^ and MERFISH is recommended to have a DV200 of >70% for fresh and fixed frozen tissue^64^ and >40% for FFPE tissue.^68^ (Note that MERFISH FFPE uses a different set of FFPE-optimized reagents from the one used for fresh and fixed frozen tissue.) It should be noted, however, that although high DV200 values in over-fixed samples can be obtained, the increase in crosslink formation caused by over-fixation might render the RNA inaccessible and reduce transcript counts.

#### Assessment of mitochondrial RNA levels

Mitochondrial RNA constitutes a substantial fraction of neuronal RNA, with 12S and 16S ribosomal RNAs. Mitochondrially encoded mRNAs are highly abundant and often persist in degraded or stressed cells. In low-quality tissue, mitochondrial RNA leakage is increased, and a mitochondrial transcript fraction exceeding ∼25% is commonly used to indicate compromised RNA integrity. Elevated mitochondrial RNA content poses a specific challenge for sequencing-based methods, because the disproportionate representation of mitochondrial reads reduces sensitivity for nuclear gene detection and biases downstream depth-dependent analyses. To mitigate this, most spatial transcriptomics platforms (e.g. 10× Visium) use poly(A) capture, excluding mitochondrial transcripts, or incorporate strategies for ribosomal RNA depletion.

#### Post-mortem interval

Tissues with a short post-mortem interval (PMI) are ideal. It has been reported extensively that with increasing PMI, there is significant degradation of RNA, leading to reductions in quality^69,70^ and alterations in reported gene expression.^69,71,72^ One study has found that the effect of PMI on RNA quality was minimal before 36 h,^50^ but after this a large decline in RNA quality was observed. Other studies have also found no correlation between PMI and RNA quality within 24,^71^ 30,^51^ or 40 h of death,^45^ although one reported no difference even at a much greater PMI of 118 h.^73^

However, a recent study found strong indications that PMI had a significant effect on gene expression.^59^ Using fresh neocortex tissues (obtained by surgical resection as part of treatment) histologically declared non-pathological by a neuropathologist, they observed selective degradation of brain activity-related genes, whereas housekeeping genes remained stable. Notably, selective enrichment for glial genes was observed, particularly in phagosome-based pathways in a time-dependent manner, with greater PMI being correlated with more extreme changes and with the most dramatic changes occurring after a PMI of 12 h. At this point, it was also demonstrated that a loss of nuclear detail on a Haematoxylin and Eosin stain and a decrease in neuronal nuclear protein (NeuN) staining occurred. Furthermore, between 4 and 12 h of PMI, the study found indications of an outgrowth of astrocyte processes. Yet, despite these changes, the total RNA electrophoresis and RIN measurements, common assessments of RNA integrity, indicated that little degradation had occurred. This is a crucial finding, because many prior analyses of the potential association of RNA quality with PMI had assessed RNA integrity using these methods or similar.

Interestingly, this was also observed between the fresh neocortex tissues obtained from epilepsy surgical resections and frozen post-mortem cortical tissues from healthy controls (PMI of 29 ± 2.6 h). In summary, this study indicates a potential confounder in post-mortem human brain studies, which should be considered when conducting ST studies on the human brain. Based on these results, <12 h of PMI is recommended; however, this might be impossible in some neuropathological cases.

#### Autofluorescence

Human brain tissue exhibits substantial autofluorescence, primarily from lipofuscin, a pigment that accumulates with age and varies across brain regions and cell types.^74,75^ Lipofuscin autofluorescence signals pose a challenge for imaging-based ST platforms such as RNAscope, potentially generating false positives in RNA spots.^74,76^ To address this, several platforms, including MERFISH and Xenium, incorporate chemical quenching or photobleaching steps into their protocols to suppress autofluorescence and minimize background interference. In workflows where these measures are not integrated, additional strategies, such as quenching with Sudan Black B^77,78^ or LED-based.^74,79^ Photobleaching has been demonstrated to reduce autofluorescence effectively and may be appropriate to implement.

#### pH

The pH of a sample is commonly used as a marker to assess brain tissue quality and is widely used by many brain banks. Although some studies found that pH was not a good predictor of RNA quality,^51,80^ most have found a correlation between more acidic pH and reduced RNA quality.^45,81-84^ There is also evidence that pH affects gene expression results.^85^ Furthermore, pH has also been shown to be strongly correlated with the degree of degradation in the cerebellar granule cell layer,^86^ with degradation occurring at a pH of ≤6.24. A neutral pH has also been found to be best for preserving ultrastructure in the post-mortem human brain,^87^ and a pH between 6 and 7 is associated with higher RNA quality.^45,80,84^ Thus, we recommend a pH between 6 and 7, preferably as close to neutral as possible.

#### Sample documentation and neuropathological assessment of donors

Brain banks perform standard neuropathological assessment of donor brains to identify the presence of pathological hallmarks associated with the most common neurological disorders. Given that most donor brains are of aged individuals, many donors are in preclinical stages of neurodegenerative disorders, and patients diagnosed with neurodegenerative disorders might have comorbidities with other illnesses. Another recommendation for brain banking initiatives is to record macroscopic information on sample origin and orientation, which will aid downstream spatial analysis. In particular, research on more complex and heterogeneous brain regions would benefit from this additional information and enable researchers to place their results in a three-dimensional context. Image registration tools developed by the big brain project, for instance, require extended macroscopic features (ventricles, nerves and cell densities) in combination with high-resolution details on cellular content. Additionally, the inclusion of information such as Braak staging would be highly valuable in the study of neurodegenerative disorders where Lewy body pathology is implicated in their progression, such as in PD and AD. Any comorbidities and relevant information, such as PMI, should also be recorded, owing to their potential impacts on transcript levels and integrity (see ‘*Agonal factors*’, ‘*Complications of neurological disorders*’ and ‘*Post-mortem interval*’).

#### Agonal factors

Although many donors might suffer from neurological disorders such as PD or AD, the cause of death in these cases, and in healthy controls, is commonly respiratory or cardiac related. However, the cause of death is an essential factor that should be considered, because it might preclude specific samples for study. Agonal factors, such as hypoxia, coma and hyperpyrexia, at the time of death have been found to have significant detrimental impacts on RNA quality in human brain tissues.^45,88^ Additionally, antemortem agonal events, such as respiratory illness, hospitalization and use of artificial respiration,^84^ in addition to a more extended agonal period,^81^ have also been associated with lower RNA quality. As such, it is advised to exclude cases with such agonal factors likely to impact RNA quality.

#### Complications of neurological disorders

Evidence suggests that neurological conditions can alter transcriptomic profiles and impact RNA quality in the post-mortem human brain. These include conditions such as AD,^89^ PD^90-92^ and epilepsy.^37^ Additionally, neurodegenerative conditions can pose a particular challenge for ST studies, because progressive degeneration of neurons and brain structures, which leads to loss of functions in these disorders, also reduces the tissue quality and, in some cases, also the RNA quality.^89^ Additionally, the presence of other comorbidities might complicate matters further. Neurodegenerative diseases, such as AD and PD, are more common amongst the elderly, yet old age is typically associated with a range of other illnesses, which are often concurrent. As such, the effect of potential comorbidities should be accounted for when undertaking human brain studies, especially those on neurodegenerative disorders. However, if these conditions are not the target of the study, it might be worth considering whether it would be appropriate to exclude these conditions to avoid potential confounders.

#### Sample size

A critical limitation of spatial transcriptomic studies in post-mortem human brain tissue is the small number of samples typically available and the cost of ST assays. Publications with at least two cases and on average ten cases per group using Visium,^8,9,11-13^ Stereo-Seq,^14,15^ MERFISH,^18,19^ CosMx^22,23^ and GeoMx^22,23^ have been applied to discover cellular organization of the human brain and pathological hallmarks. Limited sample sizes constrain statistical power, making it challenging to distinguish robust biological signals from inter-individual variability or technical noise. This is particularly relevant in human brain research, where factors such as age, sex, post-mortem interval, comorbidities and regional heterogeneity can strongly influence transcriptomic profiles. Nonetheless, spatial transcriptomics offers unique value in rare or pathologically unusual cases.

#### Selection of spatial transcriptomic platform

The capabilities and limitations of ST platforms vary greatly. Depending on the experimental design, this might affect which ST platform is most appropriate. For example, although Visium provides an unbiased capture of the whole transcriptome, its relatively low resolution and sensitivity might impact on the detail and quality of the data. This is especially true of very lowly expressed genes, which might fail to be detected by the assay. As such, it might be appropriate to use other ST platforms, such as Xenium or RNAscope, which have greater resolution and sensitivity.

Alternatively, a combination of ST platforms might be used. The unbiased whole-transcriptome ST platform, which might be lower in sensitivity or resolution, is used initially to obtain a ‘first look’ into the tissue of interest, followed by a targeted, more sensitive and higher-resolution ST platform to probe the tissue in more detail after genes of interest have been identified.^9,14,93^

Another option is to co-opt sc/snRNA-seq and match its data to the spatial transcriptome. Combining the data of the two assays could improve the resolution and sensitivity or enable transcriptome-wide profiling.^12,93^ This can be done with various software tools, such as Tangram,^94^ SpatialScope^95^ or SPOTlight.^96^ Many other tools have also been developed and reported in the literature.^97^

## Best practices for computational analysis of spatial transcriptomics data from human brain studies

ST generates rich, high-dimensional datasets that require robust computational pipelines to extract meaningful biological insights. Although this review focuses primarily on experimental and platform considerations, we now outline key analytical challenges and emerging solutions relevant to human brain studies.

### Foundational analytical workflow

ST data analysis shares many foundational steps with sc/snRNA-seq, including quality filtering, normalization, batch correction and data integration. These preprocessing steps are essential for minimizing technical noise and ensuring robust downstream interpretation.

#### Segmentation and deconvolution

Unlike dissociated single-cell data, ST platforms capture transcriptomes from intact tissue sections, necessitating segmentation to delineate individual cells or anatomical structures. This is particularly challenging in human brain tissue, where cellular morphology is diverse and mRNA localization can be distal to the nucleus.

In spot-based platforms, such as 10× Visium, each spot can contain transcripts from multiple cells, making segmentation alone insufficient. This leads to the need for computational deconvolution, which infers cell-type composition using reference sc/snRNA-seq datasets. Several methods have been developed to address this, including: Robust Cell Type Decomposition (RCTD), which leverages scRNA-seq data to estimate cell-type proportions per spot^97^; SPOTlight, which applies seeded non-negative matrix factorization (NMF)^96^; and CARD, which incorporates spatial correlation to improve accuracy in heterogeneous tissues^98^

These tools are especially valuable in post-mortem human brain studies, where cellular diversity and regional specialization complicate interpretation. Benchmarking efforts have provided practical guidelines for selection of appropriate deconvolution and spatial gene detection methods.^99,100^

#### Segmentation in high-resolution platforms

Although newer high-resolution ST platforms can achieve near single-cell or even subcellular resolution, reducing the need for deconvolution, they introduce their own analytical challenges, particularly around cell segmentation. In imaging-based platforms, segmentation typically leverages membrane and intracellular staining, often supported by machine learning algorithms. In contrast, sequencing-based platforms lack direct imaging and must infer cell boundaries from RNA signal density and spatial distribution. Methods such as Spateo,^101^ subcellular spatial transcriptomics cell segmentation (SCS)^102^ and Fast analysis of Spatial Transcriptomics (FaST)^103^ use binning strategies to aggregate neighbouring pixels or spots into putative cells. These approaches require careful calibration to avoid over- or under-segmentation. Thus, even in high-resolution platforms, segmentation remains a crucial and evolving step in the computational workflow.

#### Inter-individual variation and batch correction

Human brain studies often rely on multiple donors with significant inter-individual differences.^104^ Batch-aware models or explicit covariate correction are essential. Bayesian hierarchical frameworks can incorporate donor-level random effects, and tools such as Harmony, Seurat, scVI and Scanorama support batch correction in reference datasets prior to signature extraction.

#### Multimodal integration

Another key area of development is the integration of ST data with other modalities, such as sc/snRNA-seq, proteomics and epigenomics. Although multimodal integration enhances biological resolution, it introduces analytical complexity. Challenges include alignment of spatial data across modalities with differing resolutions, developing fusion strategies to combine transcriptomic and proteomic signals, and linking spatial gene expression patterns to anatomical or behavioural phenotypes. Frameworks such as Seurat v.5^105^ and Tangram^94^ offer promising approaches for multimodal integration, although their application to human brain tissue remains an active area of research.

#### Scalability and infrastructure

As ST studies scale to whole-brain or multi-region analyses, the volume of data generated can reach terabyte levels. Efficient data handling, parallelized processing and scalable visualization tools are essential. Platforms such as Squidpy^106^ and the Giotto Suite^107^ are increasingly adopted for large-scale spatial data analysis.

#### Standardization and future outlook

Developing scalable, accessible and reproducible computational tools is crucial for ensuring comparability across studies. Global initiatives such as the Human Cell Atlas (HCA) and the Spatial Temporal Omics Consortium (STOC) are working to establish standards for technology, metadata and analysis. These efforts will support artificial intelligence-driven modelling and translational applications, such as disease progression analysis and drug response prediction.

## Future directions

The rapid development of ST over recent years has proved it to be a disruptive and game-changing technology, heralding a new spatial frontier in human neuroscience. Analytical techniques have traditionally been centred around Western blots, RT-PCR from FF tissue or immunohistochemistry on FFPE tissue. However, the arrival of evolving technologies stemming from RNA detection has upended the existing paradigm. It might hold the key to unlocking the answers to the important biological questions of our time. Although using human brain tissues poses unique challenges, such as limited availability and variable sample integrity, these challenges are being addressed actively through improved tissue processing protocols and quality control measures. However, the future of spatial transcriptomics lies not only in overcoming sample limitations but also in embracing emerging technological innovations. Advances in spatial epigenomics, such as spatial ATAC-seq (e.g. AtlasXomics), enable researchers to map chromatin accessibility while preserving tissue architecture, offering insights into gene regulation and disease mechanisms. The AtlasXomics DBiT-seq platform supports spatial ATAC-seq and CUT&Tag assays,^108^ allowing high-resolution mapping of chromatin states and transcription factor binding directly in tissue sections. This enables identification of *cis*-regulatory elements and epigenetic signatures *in situ*, which are crucial for understanding neurodevelopment and disease progression. Likewise, *in vivo* ST adaptations, such as PERTURB-CAST, a method that streamlines measurements of perturbations at the tissue level, are being explored to allow longitudinal tracking of gene expression in live tissues, but these studies are limited to mouse models.^109^ It remains to be seen whether these can be replicated in humans.

Integration with multi-omics platforms, including spatial proteomics, are expanding the analytical depth of ST, allowing simultaneous mapping of RNA and protein expression. On the computational front, artificial intelligence-driven tools, such as Segger, Bering and Cellpose, are transforming cell segmentation and annotation. Segger,^110^ a graph neural network model, frames segmentation as a transcript-to-cell link prediction task and improves transcript assignment with high sensitivity and specificity. Bering,^111^ a deep learning model, leverages transcript colocalization patterns for joint segmentation and annotation and uses transfer learning to adapt across tissues and platforms. Cellpose,^112^a generalist segmentation model trained on diverse imaging data, provides robust segmentation across tissue types and integrates well with downstream tools. These innovations enhance resolution, sensitivity and interpretability and are crucial for identifying clinically relevant biomarkers and therapeutic targets, such as DAPK1 in AD^15^ and Lewy pathology in PD.^23^ As ST continues to evolve, integration with complementary technologies will be key to unlocking deeper insights into brain function and pathology.
